# Pre-hospital delay and its associated factors in first-ever stroke registered in communities from three cities in China

**DOI:** 10.1038/srep29795

**Published:** 2016-07-14

**Authors:** Bin Jiang, Xiaojuan Ru, Haixin Sun, Hongmei Liu, Dongling Sun, Yunhai Liu, Jiuyi Huang, Li He, Wenzhi Wang

**Affiliations:** 1 Department of Neuroepidemiology, Beijing Neurosurgical Institute, Beijing Tiantan Hospital, Capital Medical University, Beijing, China; 2 Beijing Municipal Key Laboratory of Clinical Epidemiology, Beijing, China; 3 National Office for Cerebrovascular Diseases (CVD) Prevention and Control in China, Beijing, China; 4 Department of Neurology, Xiangya Hospital, Central South University, Changsha, China; 5 Shanghai Institute of Cerebral Vascular Diseases Prevention and Cure, Shanghai, China; 6 Department of Neurology, West China Hospital, Sichuan University, Chengdu, China

## Abstract

This study aimed to explore pre-hospital delay and its associated factors in first-ever stroke registered in communities from three cities in China. The rates of delay greater than or equal to 2 hours were calculated and factors associated with delays were determined by non-conditional binary logistic regression, after adjusting for different explanatory factors. Among the 403 cases of stroke with an accurate documented time of prehospital delay, the median time (interquartile range) was 4.00 (1.50–14.00) hours. Among the 544 cases of stroke with an estimated time range of prehospital delay, 24.8% of patients were transferred to the emergency department or hospital within 2 hours, only 16.9% of patients with stroke were aware that the initial symptom represented a stroke, only 18.8% used the emergency medical service and one-third of the stroke cases were not identified by ambulance doctors. In the multivariate analyses, 8 variables or sub-variables were identified. In conclusion, prehospital delay of stroke was common in communities. Thus, intervention measures in communities should focus on education about the early identification of stroke and appropriate emergency medical service (EMS) use, as well as the development of organized stroke care.

The incidence of stroke in China is higher than in other parts of the world, and there is an increasing trend for ischemic stroke^1^,^2^. In a WHO’s Monitoring of Trends and Determinants of Cardiovascular Disease (WHO MONICA) project, the age-standardised stroke-attack rate (for first and recurrent events) in Beijing from 1985 to 1990 was 247 per 100 000 per year for men and 175 per 100 000 per year for women, sixth and second highest among the 17 MONICA populations in 10 countries, respectively^2^. The incidence of overall stroke and ICH among individuals ≥55 years of age from 1991 to 2000 was substantially higher in Beijing and Changsha than in Western populations from published literature^1^. The estimated rates of thrombolytic use for ischemic stroke are very low, ranging from only 1% to 3% in previous hospital-based studies^2^,^3^. Implementation of effective interventions to treat acute strokes within the time window, including the use of thrombolytics in acute ischemic stroke and the management of blood pressure and blood glucose during the acute period of cerebral apoplexy, has a significant impact on stroke recovery. With regard to prehospital delay in stroke, most studies are hospital-based and focus on ischemic stroke^4^. However, almost all hospital-based studies suggest that the “FAST” criteria (which include three assessment items of facial droop, arm weakness or paralysis and speech difficulty, as well as an attached message of time to promote urgency) should be implemented in communities; however, the factors identified as influencing prehospital delay of stroke were different, even contrary, across different studies. Thus, it might be inappropriate to use the evidence from hospital-based studies to conduct a “FAST” campaign on stroke in all communities.

To better direct the “FAST” compaign in communities, we aimed to explore pre-hospital delay and its associated factors in patients experiencing a first-ever stroke by examining the results from a stroke surveillance registry in communities from three cities in China.

## Methods

### Subjects

In this study, we used surveillance data of first-ever stroke in 2008 from a Chinese population of approximately 300000 residing in surburban Shanghai (SH), Changsha (CS), and Chengdu (CD). Shanghai, Changsha and Chengdu are three cities located in the eastern, central southern and western southern regions of China, respectively.

As the study initiator, the Beijing Neurosugical Institute presided over the project. The three centers that served as executors, the Shanghai Institute of Cerebral Vascular Disease Prevention and Cure, the Xiangya Hospital of Central South University in Changsha, and the West China Hospital of Sichuan University in Chengdu, were responsible for field work and data collection. Personnel from the three collabrating centers were trained by the Beijing Neurosurgical Institute before the beginning of the project.

First, three communities, each with approximately 100 000 residents, were defined in 2008 by the three aforementioned cooperation centers. Demographic information on the age and gender distributions of each community was obtained from local police stations and/or local neighborhood committees (NCs). Temporary residents and individuals who registered in the local police stations but did not actually reside in the communities were excluded.

### Stroke surveillance

The stroke report network, case ascertainment and quality control were described in detail in a previous study^1^. Briefly, at the beginning of the stroke registry in these communities, community-based stroke surveillance networks were established in all research communities to identify incident stroke cases. Doctors in local centers of community health services (CHS) or hospitals that provide basic and comprehensive medical and public health services to local residents in China were recruited as our grassroots network and trained annually by the 3 collaborating centers in this project. These doctors established close ties with the directors of local NCs and building gate volunteers who were trained to identify and report stroke cases. During the study period, the qualified doctors or general practioners (GPs) in local CHS centers or hospitals collected all the possible stroke cases during their daily health services through directors of NCs or building gate volunteers. Personnel from the 3 collaborating centers would then assess the diagnoses for all of the reported cases of stroke and death, and they were responsible for the quality of the stroke surveillance.

Detailed clinical information on each case, including age and sex, clinical signs and symptoms, past medical history, medications, computerized tomography (CT) or magnetic resonance imaging (MRI) results, and prehospital clinical status, was obtained through interviews with the patients, families, relatives or witnesses and/or retrieval from medical records in hospitals using a structured questionnaire (Supplementary Appendix 1 online). The prehospital clinical status information included date (year: month: day) and 24-hour time (hour: minute) of stroke onset and hospital arrival, stroke identification, seeking medical service behavior after symptom onset, transport means, first-visit arrival hospital and hospital grade, the first witness who noticed the symptoms or signs, other symptom onset details including nighttime or daytime onset, changes in symptoms from onset to hospital arrival, and whether the symptoms occurred on a workday or weekend (including national legal holiday). If the date and 24-hour time of stroke onset or hospital arrival could not be obtained in some cases, the estimated time range from onset to the hospital was approximated to one of 7 time bands (including <2 hours, 2 to 6 hours, 6 to12 hours, 12 to 24 hours, 1 to 3 days, 3–7 days, and >7 days) to the greatest extent possible.

Patients who did not go to the hospital or died at home were also identified as parts of the medical workers’ routines, supplemented by our annual door-to-door inquiries of the NCs directors and building gate volunteers for each building. Stroke patients who died at home were identified by eyewitnesses (patients’ relatives) and the monthly review of death certificates. In China, all deaths are legally required to be reported by patients’ relatives to the local medical workers for a death certificate used in cremation and household registration cancellation.

### Stroke Diagnosis and Classification

The diagnosis and classification of stroke were performed according to a slightly revised version of that used in the Atherosclerosis Risk in Communities (ARIC) Study^1^. Briefly, the minimum criteria for a definite or probable stroke diagnosis included evidence of sudden or rapid onset of neurological symptoms lasting for >24 hours or leading to death in the absence of evidence for a nonstroke cause. Stroke subtypes were defined according to published criteria and then grouped into 3 major types: ischemic stroke (IS, including thrombotic brain infarction, cardioembolic stroke, and symptomatic lacunar infarcts), intracerebral hemorrhage (ICH), or subarachnoid hemorrhage (SAH). Patients who fit 2 different diagnostic categories, did not go to the hospital or died at home were assigned as undetermined stroke (US).

### Definitions of studied factors

The medical histories were defined as follows: hypertension (reported systolic blood pressure ≥140 mm Hg, reported diastolic blood pressure ≥90 mm Hg, patient’s self-report of hypertension or use of antihypertensive drugs), transient ischemic attack (TIA, a temporary disturbance in brain function resulting from a temporary blockage of the brain’s blood supply that resolves within 24 hours), cardiac diseases (history of myocardial infarction, coronary artery disease, congestive heart failure, arrhythmia, or valvular heart disease), diabetes mellitus (fasting blood glucose level ≥7.8 mmol/L, patient’s self-report of diabetes, or use of antidiabetic drugs), and hyperlipidemia (reported fasting total cholesterol ≥5.72 mmol/L, reported low-density lipoprotein (LDL) ≥3.64 mmol/L, reported high-density lipoprotein (HDL) ≤0.91 mmol/L, reported triglyceride ≥1.70 mmol/L, patient’s self-report of hyperlipidemia, or use of antihyperlipidemic drugs).

The onset time was defined as the moment that a patient or witness initially noticed symptoms. When indeterminate, we defined onset time as the last time that the patient was observed without symptoms. The time of admission was defined as the time when the patients presented to the first-visit ED or hospital including primary, secondary or tertiary hospitals. Prehospital delay or time to presentation was defined as the time from symptom onset to the earliest documented time in the first visit to the emergency department or hospital.

### Emergency medical services (EMS) and organized stroke care in three cities

In China, larger cities generally use the 120 and/or 999 EMS scheduling systems. Ambulances are based at hospitals in Changsha and Chengdu or at independent facilities in Shanghai. Physicians accompany ambulances in many of the larger services on runs to severely ill patients, whereas ambulance drivers receive little or no formal training. Although there is communication between the EMS systems and hospitals in these larger cities, there is also some competition and conflict over “turf” in providing services^5^. Although the development of EMS in China has made great progress, the development of the “life green channel” for stroke is just beginning and requires further development.

Although delivering organized stroke services plays key role in the provision of effective therapies and in improving the overall outcome of stroke, organized stroke care is still far from implemented in large parts of China, and organization of prehospital care has received less attention than organized inpatient (stroke unit) care. In 2001, the first comprehensive stroke unit in China was established in Beijing. Until 2015, guidelines for the construction of a stroke center in China were first issued, and the 455 hospitals’ list of the Chinese Stroke Center Alliance (CSCA) members was announced by the Chinese Stroke Association, the Center of Medical Quality Control of the National Health and Family Planning Commission, and the CSCA. However, most hospitals in this stroke registry were not listed among the CSCA members.

### Ethics statement

This study was approved by the Ethics Committee of the Beijing Tiantan Hospital affiliated with the Capital Medical University, shared by the Beijing Neurosurgical Institute, and oral informed consent was obtained from all participants through patients’ self or caregivers. The study was performed in accordance with the Declaration of Helsinki.

### Statistical analysis

Percents were given for categorical variables, and means (±SD, median) were given for continuous variables. The prehospital delay time was presented as both a mean and a median according to a previous study’s suggestion^4^. Significant differences among groups were determined using the χ^2^ test, Fisher’s exact test, Mann-Whitney’s U-test, and analysis of variance (ANOVA).

The prehospital delay rates of greater than or equal to 2 hours were analyzed in the different subgroups, and factors associated with delays were determined using non-conditional binary logistic regression after adjusting for different explanatory factors. The explanatory risk factors included age group (<55/≥55/≥65/≥75); sex (men/women); city (Shanghai/Changsha/Chengdu); subtype of stroke (SAH/ICH/IS/US); history (yes/no/unknown) of hypertension, TIA, cardiac disease, diabetes mellitus, and hyperlipidemia; symptoms/signs (yes/no/unknown) of vomiting, headache, coma, hemiplegia, diplopia, aphasia, hemianopsia, dysesthesia, vertigo (disorders of gaits), and dysarthria (difficulty in swallowing, enunciation); the prehospital clinical status of stroke identification (yes/no/unknown); seeking medical service behavior after symptom onset (directly go to hospital by self/call 120 or 999/call for GP’s help/unknown); transport means (ambulance/private car or taxi/bicycle or tricycle or other vehicle); arrival hospital and hospital grade (tertiary hospital/secondary hospital/primary hospital or CHS center); first witness who noticed symptoms or signs (patients’ self/family/field witness/GPs or unknown); onset details including nighttime or daytime onset (nighttime/daytime/unknown); changes in symptoms from onset to hospital presentation (worsened/completely improved/partially improved/unchanged/unknown); and workday or weekend (workdays/holidays). In the multivariate analysis, the estimated time range of prehospital delay was used. Given collinearity and problems with multivariable results from small numbers in some categories in the multivariate analysis, the multivariable analysis was done using forward stepwise regression with likelihood ratio to obtain a more parsimonious and reliable model. A sensitivity analysis for the multivariable analysis was also done to validate the results using patients with an accurate time of prehospital delay. All statistical calculations were performed using SPSS 13.0 software (SPSS Inc. Chicago, IL, USA). P < 0.05 was considered statistically significant.

### Role of the funding source

The funder had no role in the study design, data collection, analysis, interpretation, or the writing of the report. The project investigators were responsible for the decision to submit the report for publication.

## Results

### Stroke registration

Altogether, 666 cases of first-ever stroke (3 SAH; 47 ICH; 175 IS; 2 US in SH, 4 SAH; 98 ICH; 109 IS; 7 US in CS, and 8 SAH; 43 ICH; 160 IS; 10 US in CD) were registered from a population of 341,207 individuals (100,622 people in SH, 110,411 people in CS, and 130,174 people in CD). Among the 666 cases, 122 cases were excluded because of missing information on prehospital clinical status including prehospital delay. Therefore, 544 cases of the 666 cases had an estimated time range of prehospital delay, and 403 cases (218 cases in SH, 93 cases in CS, and 92 cases in CD) of the 544 cases also had an accurate time of prehospital delay. Among the 544 cases, 220 cases (3 SAH; 46 ICH; 170 IS; 1 US) were registered from 12 hospitals (5 tertiary hospitals, 3 secondary hospitals and 4 primary hospitals) in SH, 178 cases (3 SAH; 81 ICH; 87 IS; 7 US) from 9 hospitals (5 tertiary hospitals, 2 secondary hospitals and 2 primary hospitals) in CS, and 146 cases (6 SAH; 28 ICH; 109 IS; 3 US) from 9 hospitals (2 tertiary hospitals, 5 secondary hospitals and 2 primary hospitals) in CD. No significant differences were noted between all of the stroke cases and cases with an accurate time or estimated time range of prehospital delay regarding the variables of age, sex, and stroke subtype (Table 1). In other words, the cases with an accurate time or estimated time range of prehospital delay represented all stroke cases.

### Characteristics and clinical status of pre-hospital transfers among patients with stroke registered in the three cities

Table 2 shows that characteristics and clinical status of pre-hospital transfers among patients with stroke registered in the 3 Chinese communities. Among the 544 cases of stroke, only 16.9% of patients with stroke were aware that the initial symptom was a stroke (by the patient/bystander), and 18.8% used the EMS (one third of the strokes were not identified by the ambulance doctors). In the first-visit to the emergency department or hospital, 4.2% of patients arrived at CHS centers or local hospitals, 71.5% of patients were transferred to a secondary hospital, and 24.3% of patients were transferred to a tertiary hospital. Among the 403 cases of stroke, the median (interquartile range) value was 4.00 (1.50–14.00) hours. In the communities studied, 24.8% of patients from the 544 cases were transferred to the emergency department or hospital within 2 hours, while 49.1% of the patients from the 399 cases were transferred within 3 hours.

### Associated factors for prehospital delay

In univariate analyses, 17 variables or sub-variables were associated with prehospital delay ≥2 hours, whereas 8 variables or sub-variables were identified in the multivariate analyses. The proportions of prehospital delay ≥2 hours in patients with stroke in CS and CD were higher, respectively 3.6 times and 2.2 times, than in SH, after adjusting for other variables in the multivariate analyses. The proportion of prehospital delay ≥2 hours in patients with stroke whose symptoms/signs were discovered by a cohabitant or non-cohabitant witness was lower, respectively 1/2 and 1/4, compared to the cases identified by the patient. The proportion of prehospital delay ≥2 hours in patients who had symptoms of coma was lower than that in those who did not. The proportion of prehospital delay ≥2 hours in patients with a stroke that occurred at nighttime was higher in comparison to those with daytime stroke; the proportion of prehospital delay ≥2 hours in patients who were unaware of their stroke symptoms was higher than that in patients who were aware; and the proportion of prehospital delay ≥2 hours in those whose symptoms were partially improved was higher than that in patients whose symptoms were unchanged (Table 3). In the sensitivity analysis, more consistent results from patients with an accurate time of prehospital delay and with an estimated time range of prehospital delay were found except “coma” variable (data not shown).

## Discussion

In this investigation, half of the patients with a stroke were transferred to an emergency department or hospital within 3 hours; the median (interquartile range) delay was 4.00 (1.50–14.00) hours. Both the proportion and the median delay were better than 25% and 15.0 (2.8–51.0) hours in a previous report^3^. The present study was distinctly different from this previous hospital-based study, which involved 62 hospitals across a variety of economic and geographic regions in China during 2006; it mainly focused on prehospital delay in first visits to the emergency department or hospital in a community-based surveillance registry of patients experiencing a first-ever stroke in 3 large cities. The median prehospital delay in this study ranged between 3 and 4 hours, which was the median delay of symptom onset to ED arrival reported in previous studies published since the year 2000^4^.

The factors associated with prehospital delay are numerous and include socio-demographic characteristics, clinical factors, contextural/social factors, cognitive factors and behavioral factors. For each factor, different associations were found across different studies. Although older age was found to be associated with prehospital delay in previous studies^6^,^7^,^8^,^9^,^10^,^11^,^12^, no association between older age and prehospital delay was found in our study, similar to other studies^13^,^14^,^15^,^16^,^17^,^18^,^19^,^20^,^21^,^22^,^23^,^24^,^25^,^26^,^27^,^28^,^29^,^30^,^31^. Contrary association with prehospital delay for stroke patients between sexes was found in some studies^16^,^19^,^23^,^25^, but no association was also found in our study, similar to most studies^6^,^7^,^8^,^9^,^10^,^13^,^14^,^15^,^17^,^18^,^20^,^21^,^22^,^24^,^26^,^27^,^28^,^29^. Regarding regional differences, the differences in the proportions of prehospital delay ≥2 hours between CS, CD and SH were probably due to different EMS deliveries.

No difference in prehospital delay between haemorrhagic and ischemic stroke was found in our multivariate analysis, consistent with most other studies^6^,^7^,^8^,^14^,^16^,^18^,^19^,^24^,^29^,^30^. Interestingly, the proportion of patients with hemorrhagic stroke in the present study was relatively higher in the CS region, as noted in a previous stroke registry^32^. Further, the proportion of hemorrhagic stroke patients with a prehospital delay ≥2 hours was higher than that for ischemic stroke patients in CS, in contrast to the findings in SH and CD.

No association between co-morbidities and prehospital delay for stroke was found, which is in agreement with the results from most prior studies^8^,^9^,^10^,^14^,^16^,^17^,^18^,^19^,^21^,^23^,^26^,^28^,^30^,^31^. Regarding symptoms and/or signs, only patients with coma were found to be less likely to have a prehospital delay compared to patients without. Similar to this study, stroke patients with coma were often transferred to the emergency department or hospital earlier in previous studies^11^,^15^,^26^,^28^,^33^.

In the present study, patients whose stroke symptoms were first noticed by cohabitants and non-cohabitant witnesses arrived at the hospital earlier, than patients who first noticed the stroke symptoms themselves. These findings could be potentially explained by different coping patterns: the patients themselves may have had a passive coping pattern after recognizing the symptoms of stroke, whereas witnesses often took a more active approach.

Previous studies have shown that patients whose symptoms improved between the symptom onset and hospital arrival were less likely to experience a prehospital delay^34^, whereas patients whose symptoms worsened were more likely to experience a prehospital delay^11^,^28^,^35^. However, the present study showed that only patients with partially improved symptoms, had an increased likelihood of prehospital delay when compared to patients with unchanged symptoms.

In the present study, patients with a nighttime onset of symptoms were more likely to have a prehospital delay compared to patients with a daytime onset. In other studies, patients whose symptoms presented during the day arrived at the emergency department or hospital earlier^36^, consistent with this study, and patients with nighttime-onset stroke were more likely to have a prehospital delay for stroke evaluation^13^,^20^. Although nighttime onset was not found to be associated with prehospital delay in most previous studies^10^,^17^,^18^,^19^,^24^, similar to daytime onset^7^,^8^,^17^,^18^,^22^,^25^,^30^, one study even suggested that nocturnal onset independently contributed to early arrival^26^.

One study found that a Sunday onset was associated with prehospital delay of stroke within 1 hour^29^. However, no difference in prehospital delay was found between patients who developed symptoms on workdays versus weekends in the present study.

Although no influence of stroke awareness on prehospital delay was found in many previous studies^7^,^10^,^19^,^23^,^24^,^29^,^34^,^37^, other studies did find that when patients or bystanders were aware that the initial symptom was a stroke, the patient often arrived at the emergency department or hospital earlier than patients who were unaware^26^,^27^,^38^. The awareness rate of stroke warning symptoms ranged from 58.2% to 80.2% in stroke-free populations from these three cities in a previous study^39^. However, in the present study, only 16.9% of patients were aware that they were having a stroke. The proportion of prehospital delay ≥2 hours in stroke patients without awareness was higher than that in stroke patients with awareness. These findings suggest that the reported knowledge of early stroke symptoms in people who have not experienced a stroke is not equivalent to the patients’/bystanders’ ability to recognize the initial symptoms of a stroke when it is occurring. In addition, not all patients who were aware that their initial symptom was a stroke reported to EMS to achieve rapid transfer, although the proportion of patients who were aware of their stroke symptom and contacted EMS was higher than that in patients who were unaware.

In the present study, only 18.8% of patients used the emergency medical aid service, and one-third of the stroke cases were not identified by ambulance doctors. According to the present investigation, the distance from the site of symptom onset to the hospital was obviously not the cause of prehospital delay for stroke, as 92.5% of patients with first-ever stroke were transferred to the emergency department or hospitals within 2 hours, and no association was found between prehospital delay of stroke and means of prehospital transfer. In fact, one-quarter of patients arrived at the emergency department or hospital within 2 hours but did not receive standard treatment. Therefore, it would be beneficial to improve EMS for stroke in China to address the conflicts of interest between the hospital and the EMS system, improve the acute care of the “life green channel” for stroke in the hospital, and achieve more rapid interactions between prehospital transfer in the EMS system and the “life green channel” for stroke in the hospital through implementation of organized stroke care.

The results of the univariate analysis of this study, but not the multivariate analysis, found that the use of EMS services reduced prehospital delay when compared to patients who arrived at the hospital by themselves, and these finding are consistent with most previous studies^6^,^8^,^12^,^16^,^19^,^20^,^21^,^22^,^23^,^25^,^27^,^28^,^29^,^30^,^36^,^38^,^40^. These contradictory analyses may be due to a limited study sample; it is also possible that validity may be difficult to maintain a significant difference in OR values, or that the development of private car or taxi services in these larger cities may invalidate the results. In contrast, prehospital delay of arrival at the emergency department or hospital by non-EMS transfer was longer, as documented in previous studies^11^,^35^.

Another study showed that prehospital delay of arrival at a teaching hospital was longer, as these cases constituted non-first-referral at a teaching hospital^20^. However, the proportion of prehospital delay ≥2 hours at secondary hospitals was lower than that at tertiary hospitals in our univaritate analysis, although no difference in prehospital delay was found in the multivariate analysis. The present study evaluated information from a first-ever stroke registry regarding prehospital delay for patients’ first visits to the hospital, thus, our findings did not pertain to non-first-referral patients. Further analysis showed that the proportion of transfer time on the road of first visits to a secondary hospital within 2 hours was significantly higher than that to a tertiary hospital. Differences in the prehospital delay between different grade hospitals were likely due to resource allocation of different grade hospitals in communities, which led to differences in arrival times to different- grade hospitals.

In the present study, only 4.2% of patients arrived at local CHS centers or hospitals, and 4 patients with stroke waited for their GPs at home in SH. Thus, GP contact was not a risk factor for prehospital delay in this target populations, which was different from the findings in other studies^7^,^24^,^34^. Obviously, it is necessary to improve the knowledge of stroke warning symptoms and the awareness of EMS use in populations through health education. Indeed, the proportion of patients who arrived at local hospitals or CHS centers was not high in our investigation, and our findings further showed that patients lacked an awareness of the rapid transfer following stroke to qualified hospitals for acute stroke care. In brief, the continuity of optimal stroke care calls for the development of organized stroke care, covering organization of prehospital care, hospital treatment and follow-up care (rehabilitation and recurrence prevention) from available health resources and services in China.

The strength of this study was its use of a community-based stroke registry that collected information on first visits to an emergency department or hospital without organized stroke care. In particular, these findings are more suitable for education regarding the “FAST” compaign in communities in the developing countries where organized stroke care is nonexistent. However, it was difficult to completely register stroke information on presentation to the emergency or hospitals in communities. Nonetheless, no differences in age, sex, or major subtypes of stroke were found, which implied that the patient sample used in this analysis could be representative of all strokes registered in communities.

In summary, prehospital delay for stroke was common in communities and was attributed to a lack of awareness of the symptoms of stroke, not calling for emergency medical services, and the lack of effective interactions between prehospital transfer and the “life green channel” for stroke in hospitals. Thus, intervention measures in communities should focus on education regarding the early identification of stroke, the importance of calling for EMS, and the development of organized stroke care.

## Additional Information

**How to cite this article**: Jiang, B. *et al*. Pre-hospital delay and its associated factors in first-ever stroke registered in communities from three cities in China. *Sci. Rep.* **6**, 29795; doi: 10.1038/srep29795 (2016).

## Supplementary Material

Supplementary Information

## Figures and Tables

**Table 1:** Differences between total cases and cases with accurate time or estimated time range of prehospital delay based on age, sex, and stroke subtype.

	Registered case (n = 666)	Cases with accurate time of prehospital delay (n = 403)	P value	Cases with estimated time range of prehospital delay (n = 544)	P value
Age (year)	70.55 ± 11.98	70.90 ± 11.77	0.642	70.31 ± 11.84	0.721
Sex					
Men	341	189	0.173	270	0.587
Women	325	214		274	
Subtype of stroke					
SAH	15	7	0.900	12	0.835
ICH	188	111		155	
IS	444	275		366	
US	19	10		11	

Note: IS, ischemic stroke; ICH, intracerebral hemorrhage ; SAH, subarachnoid hemorrhage; US, undetermined stroke.

**Table 2:** Characteristics and the clinical status of prehospital transfers among patients with stroke registered in communities from 3 cities.

	Total (n = 544)	Shanghai (n = 220)	Changsha (n = 178)	Chengdu (n = 146)	P value
Sex					
Men	49.6%	48.6%	50.0%	50.7%	0.922
Women	50.4%	51.4%	50.0%	49.3%	
Age group					
<55	11.4%	6.4%	18.0%	11.0%	<0.001
≥55	17.3%	20.5%	19.1%	10.3%	
≥65	32.5%	26.8%	40.4%	31.5%	
≥75	38.8%	46.4%	22.5%	47.3%	
Subtype of stroke					
SAH	2.2%	1.4%	1.7%	4.1%	<0.001
ICH	28.5%	20.9%	45.5%	19.2%	
IS	67.3%	77.3%	48.9%	74.7%	
US	2.0%	0.5%	3.9%	2.1%	
History					
Hypertension					
Yes	69.1%	84.1%	48.3%	71.9%	<0.001
No	23.2%	13.2%	32.6%	26.7%	
Unknown	7.7%	2.7%	19.1%	1.4%	
Diabetes					
Yes	20.7%	7.3%	38.8%	19.2%	<0.001
No	71.7%	90.9%	42.1%	78.8%	
Unknown	7.5%	1.8%	19.1%	2.1%	
Hyperlipidemia					
Yes	12.7%	4.5%	12.4%	25.3%	<0.001
No	69.9%	85.9%	52.2%	67.1%	
Unknown	17.5%	9.5%	35.4%	7.5%	
Cardiac disease					
Yes	21.0%	17.3%	18.5%	29.5%	<0.001
No	68.2%	78.2%	59.0%	64.4%	
Unknown	10.8%	4.5%	22.5%	6.2%	
TIA					
Yes	9.0%	10.9%	8.4%	6.8%	<0.001
No	77.2%	87.7%	56.7%	86.3%	
Unknown	13.8%	1.4%	34.8%	6.8%	
Symptoms/signs					
Vomiting					
Yes	25.4%	25.5%	30.3%	19.2%	<0.001
No	68.9%	73.6%	53.9%	80.1%	
Unknown	5.7%	0.9%	15.7%	0.7%	
Coma					
Yes	23.3%	22.7%	17.4%	31.5%	<0.001
No	70.8%	77.3%	64.6%	68.5%	
Unknown	5.9%	—	18.0%	—	
Hemiplegia					
Yes	59.7%	73.2%	57.9%	41.8%	<0.001
No	34.4%	20.9%	32.0%	57.5%	
Unknown	5.9%	5.9%	10.1%	0.7%	
Diplopia					
Yes	2.2%	0.9%	5.1%	0.7%	<0.001
No	86.0%	90.5%	73.0%	95.2%	
Unknown	11.8%	8.6%	21.9%	4.1%	
Aphasia					
Yes	14.3%	16.8%	9.6%	16.4%	<0.001
No	74.8%	75.5%	67.4%	82.9%	
Unknown	10.8%	7.7%	23.0%	0.7%	
Dysarthria					
Yes	18.6%	18.6%	14.0%	24.0%	<0.001
No	70.6%	73.2%	64.0%	74.7%	
Unknown	10.8%	8.2%	21.9%	1.4%	
Headache					
Yes	22.6%	21.8%	23.6%	22.6%	<0.001
No	69.7%	75.5%	56.7%	76.7%	
Unknown	7.7%	2.7%	19.7%	0.7%	
Hemianopsia					
Yes	0.7%	—	1.1%	1.4%	<0.001
No	86.0%	89.1%	75.3%	94.5%	
Unknown	13.2%	10.9%	23.6%	4.1%	
Dysesthesia					
Yes	27.6%	41.8%	14.6%	21.9%	<0.001
No	62.5%	53.2%	61.8%	77.4%	
Unknown	9.9%	5.0%	23.6%	0.7%	
Vertigo					
Yes	26.8%	36.4%	16.9%	24.7%	<0.001
No	63.4%	56.8%	62.4%	74.7%	
Unknown	9.7%	6.8%	20.8%	0.7%	
Hospital grade at initial arrival					
Tertiary	24.2%	2.7%	52.8%	21.9%	<0.001
Secondary	71.5%	90.9%	43.8%	76.0%	
Primary or CHS centers or stations	4.2%	6.4%	3.4%	2.1%	
Nighttime or daytime onset					
Nighttime	15.6%	15.5%	16.9%	14.4%	0.002
Daytime	80.1%	84.5%	76.4%	78.1%	
Unknown	4.2%	—	6.7%	7.5%	
First witness					
Patient’s self	50.7%	50.9%	47.2%	54.8%	<0.001
Family	34.9%	29.1%	39.3%	38.4%	
Field witness	11.4%	20.0%	6.7%	4.1%	
Unknown (/GPs)	2.9%	—	6.7%	2.7%	
Stroke identification					
Yes	16.9%	21.8%	19.1%	6.8%	<0.001
No	37.1%	34.1%	57.3%	17.1%	
Don’t know what the disease	46.0%	44.1%	23.6%	76.0%	
Seeking medical service behavior after symptom onset					
Go to hospital by patient’s self	79.0%	88.2%	79.8%	64.4%	<0.001
Call 120 or 999	19.5%	9.1%	19.1%	35.6%	
Wait for GPs	0.7%	1.8%	—	—	
unknown	0.7%	0.9%	1.1%	—	
Transport means					
Ambulance (120, 999)	18.8%	9.1%	19.1%	32.9%	<0.001
Private car or taxi	77.6%	87.7%	77.0%	63.0%	
Bicycle, tricycle or other	3.7%	3.2%	3.9%	4.1%	
Changes in symptoms from onset to hospital presentation					
Worsened	39.3%	43.2%	30.3%	44.5%	<0.001
Completely improved	1.8%	—	5.6%	—	
Partially improved	15.4%	3.6%	24.7%	21.9%	
Unchanged	39.9%	49.5%	33.7%	32.9%	
Unknown	3.5%	3.6%	5.6%	0.7%	
workday or weekend					
Holidays	31.3%	33.6%	30.3%	28.8%	0.585
Workdays	68.8%	66.4%	69.7%	71.2%	
Transfer time on the road					
<2 hours	92.5%	98.2%	92.7%	83.6%	<0.001
≥2 hours	7.5%	1.8%	7.3%	16.4%	
Estimated time range of Prehospital delay					
<2 hours	24.8%	39.5%	10.1%	20.5%	<0.001
2–6 hours	25.6%	27.3%	29.2%	18.5%	
6–12 hours	15.4%	8.2%	29.2%	9.6%	
12–24 hours	7.5%	15.0%	1.1%	4.1%	
1–3 days	13.4%	5.9%	18.0%	19.2%	
3–7 days	9.4%	2.7%	10.7%	17.8%	
>7 days	3.9%	1.4%	1.7%	10.3%	
Accurate prehospital delay in 403 stroke patients					
All Mean ± SD Median (IQR)	403 18.24 ± 34.24 4.00 (1.50–14.00)	218 11.57 ± 26.85 2.00 (1.50–11.63)	93 27.31 ± 41.60 7.00 (3.63–44.74)	92 24.85 ± 38.44 5.17 (1.15–36.25)	<0.001
IS Mean ± SD Median (IQR)	275 18.42 ± 34.59 4.00 (1.50–14.00)	168 13.87 ± 29.91 2.50 (1.50–13.00)	40 22.25 ± 41.57 6.00 (3.06–9.50)	67 27.57 ± 39.20 6.17 (1.31–54.67)	0.022
ICH Mean ± SD Median (IQR)	111 17.82 ± 34.40 2.52 (1.50–10.17)	46 4.11 ± 8.38 1.50 (1.00–2.50)	47 31.67 ± 43.17 7.98 (4.00–53.52)	18 16.72 ± 37.24 2.00 (1.00–10.04)	<0.001

Note: IS, ischemic stroke; ICH, intracerebral hemorrhage ; SAH, subarachnoid hemorrhage; US, undetermined stroke; TIA, transient ischemic attack; CHS, community health services; GPs, general practioners; SD, standard deviation; IQR, interquartile range.

**Table 3:** Proportions of prehospital delays ≥2 hours, shown with significant crude and adjusted odds ratios and 95% confidence intervals, in different target subjects (N = 544).

	N	% (N/544)	n (≥2 hrs)	% (n/N)	Crude OR	95% CI	Adjusted OR	95% CI
City								
Shanghai	220	40.4%	133	60.5%	Reference	Reference	Reference	Reference
Changsha	178	32.7%	160	89.9%	5.815	3.331–10.151	3.578	1.844–6.944
Chengdu	146	26.8%	116	79.5%	2.529	1.559–4.104	2.175	1.206–3.923
Subtype of stroke								
SAH	12	2.2%	8	66.7%	0.559	0.164–1.905	No data	No data
ICH	155	28.5%	108	69.7%	0.643	0.421–0.981	No data	No data
IS	366	67.3%	286	78.1%	Reference	Reference	No data	No data
US	11	2.0%	7	63.6%	0.490	0.140–1.714	No data	No data
History								
Hypertension								
Yes	376	69.1%	269	71.5%	0.503	0.299–0.845	No data	No data
No	126	23.2%	105	83.3%	Reference	Reference	No data	No data
Unknown	42	7.7%	35	83.3%	1.000	0.392–2.552	No data	No data
Diabetes								
Yes	113	20.8%	99	87.6%	2.885	1.582–5.261	No data	No data
No	390	71.7%	277	71.0%	Reference	Reference	No data	No data
Unknown	41	7.5%	33	80.5%	1.683	0.754–3.755	No data	No data
Symptoms/signs								
Vomiting								
Yes	138	25.4%	89	64.5%	0.524	0.343–0.802	No data	No data
No	375	68.9%	291	77.6%	Reference	Reference	No data	No data
Unknown	31	5.7%	29	93.5%	4.186	0.979–17.903	No data	No data
Coma								
Yes	127	23.3%	73	57.5%	0.355	0.231–0.545	0.454	0.266–0.775
No	385	70.8%	305	79.2%	Reference	Reference	Reference	Reference
Unknown	32	5.9%	31	96.9%	8.131	1.093–60.473	2.724	0.313–23.749
Hemiplegia								
Yes	325	59.7%	236	72.6%	0.632	0.408–0.979	No data	No data
No	187	34.4%	151	80.7%	Reference	Reference	No data	No data
Unknown	32	5.9%	22	68.7%	0.525	0.228–1.204	No data	No data
Headache								
Yes	123	22.6%	82	66.7%	0.623	0.400–0.970	No data	No data
No	379	69.7%	289	76.3%	Reference	Reference	No data	No data
Unknown	42	7.7%	38	90.5%	2.958	1.028–8.514	No data	No data
Vertigo								
Yes	146	26.8%	120	82.2%	1.831	1.128–2.972	No data	No data
No	345	63.4%	247	71.6%	Reference	Reference	No data	No data
Unknown	53	9.7%	42	79.2%	1.515	0.749–3.062	No data	No data
Hospital grade at initial arrival								
Tertiary	132	24.3%	113	85.6%	Reference	Reference	No data	No data
Secondary	389	71.5%	280	72.0%	0.432	0.253–0.737	No data	No data
Primary or CHS centers or stations	23	4.2%	16	69.6%	0.384	0.140–1.058	No data	No data
Nighttime or daytime onset								
Nighttime	85	15.6%	75	88.2%	2.914	1.458–5.822	3.264	1.525–6.988
Daytime	436	80.1%	314	72.0%	Reference	Reference	Reference	Reference
Unknown	23	4.2%	20	87.0%	2.590	0.756–8.874	2.655	0.676–10.422
First witness								
Patient’s self	276	50.7%	233	84.4%	Reference	Reference	Reference	Reference
Family	190	34.9%	139	73.2%	0.503	0.319–0.794	0.541	0.318–0.920
Field witness	62	11.4%	24	38.7%	0.117	0.064–0.214	0.254	0.125–0.516
Unknown (/GPs)	16	2.9%	13	81.2%	0.800	0.219–2.925	0.456	0.102–2.040
Stroke identification								
Yes	92	16.9%	65	70.7%	Reference	Reference	Reference	Reference
No	202	37.1%	174	86.1%	2.581	1.416–4.706	2.130	1.080–4.200
Don’t know what the disease	250	46.0%	170	68.0%	0.883	0.524–1.487	1.011	0.583–1.900
Seeking medical service behavior after symptom onset								
Go to hospital by patient’s self	430	79.0%	333	77.4%	Reference	Reference	No data	No data
Call 120 or 999 for ambulance	106	19.5%	71	67.0%	0.591	0.372–0.939	No data	No data
Wait for GPs	4	0.7%	2	50.0%	0.291	0.041–2.095	No data	No data
unknown	4	0.7%	3	75.0%	0.874	0.090–8.496	No data	No data
Changes in symptoms from onset to hospital presentation								
Worsened	214	39.3%	156	72.9%	1.150	0.757–1.748	1.154	0.711–1.874
Completely improved	10	1.8%	9	90.0%	3.849	0.478–31.001	0.829	0.088–7.832
Partially improved	84	15.4%	81	96.4%	11.546	3.518–37.892	7.075	2.022–24.760
Unchanged	217	39.9%	152	70.0%	Reference	Reference	Reference	Reference
Unknown	19	3.5%	11	57.9%	0.588	0.226–1.529	0.533	0.167–1.702

Note: OR, odd ratio; 95% CI, 95% confidence interval; IS, ischemic stroke; ICH, intracerebral hemorrhage ; SAH, subarachnoid hemorrhage; US, undetermined stroke; TIA, transient ischemic attack; CHS, community health services; GPs, general practioners.
