# Supplementary material for: Pre-hospital delay and its associated factors in first-ever stroke registered in communities from three cities in China
Source: Sci Rep. 2016 Jul 14;6:29795. doi: 10.1038/srep29795 (PMC4944187; doi:10.1038/srep29795)
Supplement: Supplementary Information [file srep29795-s1.doc]

**Pre-hospital delay and its associated factors in first-ever stroke registered in communities from three cities in China**

Bin JIANG*, Xiaojuan RU, Haixin SUN, Hongmei LIU, Dongling SUN, Yunhai LIU, Jiuyi HUANG, Li HE, Wenzhi WANG*

Department of Neuroepidemiology, Beijing Neurosurgical Institute, Beijing Tiantan Hospital, Capital Medical University, Beijing, China (Prof B Jiang MD, X Ru MD, H Sun MD, H Liu MD, D Sun, Prof W Wang MD),

Beijing Municipal Key Laboratory of Clinical Epidemiology, Beijing, China (Prof B Jiang MD, X Ru MD, H Sun MD, H Liu MD, D Sun, Prof W Wang MD),

Department of Neurology, Xiangya Hospital, Central South University, Changsha, China (Prof Y Liu MD),

Shanghai Institute of Cerebral Vascular Diseases Prevention and Cure, Shanghai, China (Prof J Huang MD),

Department of Neurology, West China Hospital, Sichuan University, Chengdu, China (Prof L He MD),

National Office for Cerebrovascular Diseases (CVD) Prevention and Control in China, Beijing, China (H Liu MD, Prof W Wang MD)

*Correspondence to:

Prof Bin Jiang,

Department of Neuroepidemiology,

Beijing Neurosurgical Institute,

Beijing Tiantan Hospital,

Capital Medical University,

6 TiantanXili, Dongcheng District

Beijing 100050, P. R. China

E-mail: [bjyjiang@hotmail.com](mailto:bjyjiang@hotmail.com), [bjyjiang@163.com](mailto:bjyjiang@163.com)

Or

Prof Wenzhi Wang,

National Office for Cerebrovascular Diseases (CVD) Prevention and Control in China,

Beijing Neurosurgical Institute,

Beijing Tiantan Hospital,

Capital Medical University,

6 TiantanXili, Dongcheng District

Beijing 100050, P. R. China

E-mail: [qgnfbwwz@163.com](mailto:qgnfbwwz@163.com)

**Supplementary information:**

Supplementary Appendix 1: Table A-2 Registration Form for nonfatal or fatal stroke

**Table A-2 Registration Form for nonfatal or fatal stroke**

（All first-ever, recurrent and fatal stroke should be registered and filled in）

**A.General information**

| A1**.** Collaborating center code： □□  1= Shanghai Institute of Cerebral Vascular Disease Prevention and Cure 2= Xiangya Hospital 3= West China Hospital  A2**.** Community code：1=Intervention 2=Control□ | A3**.** CHS/Local primary hospital:________________ Code: □□  A4**.** Neighborhood committee：__________________ Code: □□  A5.Case No.： □□□□  A6.Sex： 1=Men 2=Women □ | |
| --- | --- | --- |
| A7.Name：_____________________A8.Address：_____________________________________________ | | |
| A9.Birth date： | | □□□□Year/□□Month/□□Day |

**B. Information on this incident stroke**

| course of disease | Brief description, including predisposing factors, status during disease onset, disease progression, and results of special examinations. (If possible, record the name of the hospital, case number or numbers of CT/MRI, EMG and review these reports.): | | |
| --- | --- | --- | --- |
|  | | |
|  | | |
|  | | |
|  | | |
|  | | |
| B1 | Case sourse：  1=Case from admission 2=Case from outpatient or emergency department 3=Community Health Center (station) 4=At home | | □ |
| B2 | Onset date： | □□□□ Year /□□Month /□□Day | |
| B3 | Visit date： | □□□□ Year /□□Month /□□Day | |
| B4 | Diagnostic doctor：  1=Neurologist 2=Non neurological physician 3=other（___________________________） | | □ |
| B5 | Visit hospital or visit information：  1= Hospital of district, county level or above 2=Local primary hospital 3=diagnosed by physician at home 4=untreated 5=unknown | | □ |
| B6 | This incident stroke：1=First-ever stroke(Skip to C6) 2=Recurrent stroke | | □ |

**C. Anamnesis**：

| C1 | Date of first-ever strke： | | | | □□□□ Year /□□Month /□□Day | | | |
| --- | --- | --- | --- | --- | --- | --- | --- | --- |
| C2 | Subtype of first-ever stroke：  1=SAH 2=ICH 3=Ischemic stroke 4=Other（_____________________） | | | | | | | □ |
| C3 | Date of recent last stroke： | | | | □□□□ Year /□□Month /□□Day | | | |
| C4 | Subtype of recent last stroke：  1=SAH 2=ICH 3=Ischemic stroke 4=Other（_____________________） | | | | | | | □ |
| C5 | Which time was this incident stroke?＿＿ | | | | | | □□ | |
| C6 | | Other disease history：1=Yes 2=No 9=Unknown | | | | | | |
|  |  | Diabetes | □C6-1 | Dyslipidemia | | □C6-4 | | |
|  |  | hypertension | □C6-2 | Cardiac disease | | □C6-5 | | |
|  |  | TIA | □C6-3 |  | |  | | |

**D.Neurological symptoms and signs：1=Yes 2=No 9=Unknown**

| Vomiting | □D-1 | Severe headache | □D-7 |
| --- | --- | --- | --- |
| Coma | □D-2 | Hemianopsia | □D-8 |
| Hemiplegia | □D-3 | Dysesthesia | □D-9 |
| Diplopia | □D-4 | Meningeal irritation sign | □D-10 |
| Aphasia | □D-5 | Vertigo or gait disturbance | □D-11 |
| Dysarthria | □D-6 | Other（________________） | □D-12 |

**E. Special examination：1=Yes 2=No 9=Unknown**

| CT | □E-1 | Lumbar puncture | □E-7 |
| --- | --- | --- | --- |
| Magnetic resonance（MRI） | □E-2 | Single photon emission computerized tomogr（SPECT） | □E-8 |
| Autopsy | □E-3 | Transcranial Doppler ultrasound (TCD) | □E-9 |
| Angiography | □E-4 | Other（________________） | □E-10 |

**F. Subtype of stroke：（Initial diagnosis or preliminary diagnosis） □□**

| Code of diagnosis | 1=Definite SAH  3=Definite ICH  5=Definite thrombatic brain infarction  7=Definite cardioembolic stroke  9=Illdefined complete stroke  11=Lacunar stroke | 2=Probable SAH  4=Probable ICH  6= Probable thrombatic brain infarction  8= Probable cardioembolic stroke  10=Undocumented fatal stroke |
| --- | --- | --- |

**G.Checked diagnosis**：

| G1 | 1=Same as initial diagnosis 2=Different from initial diagnosis | □ |
| --- | --- | --- |
| G2 | If different from intial diagnosis，should be changed as：________ | □□ |
|  | Basis of modified diagnosis：____________________________________________________________ | |

Reporter：＿＿＿＿＿＿ Reporting date：＿＿＿＿＿＿＿＿

Auditing physician：＿＿＿＿＿＿ Auditing date：＿＿＿＿＿＿＿＿

**H.If death cases，need to fill in the following information**：

| H1 | Death date： | □□□□ Year /□□Month /□□Day |
| --- | --- | --- |
| H2 | | Chief disease history, description of death process (including any predisposing factors, clinical manifestations, disease progression, and hospital where the diagnosis was made): | | --- | | |
|  | |
|  | |
|  | |
|  | |
|  | |

| H3 | Death diagnosis： | |
| --- | --- | --- |
|  | Underlying disease：________________________________________ | ICD code□-□□ |
|  | Disease directly associated with death：_________________________ | ICD code□-□□ |

| H4 | Level of the hospital where the diagnosis was made：  1=Provincial/municipal hospital or higher 2=District/county hospital 3=Local hospital lower than level of district/county hospital  4=Untreated（indicate who made the diagnosis__________________________） | □ |
| --- | --- | --- |
| H5 | Information on relationship between provider and the dead：__________________________ | |

Reporter：＿＿＿＿＿＿ Reporting date：＿＿＿＿＿＿＿＿

Auditing physician：＿＿＿＿＿＿ Auditing date：＿＿＿＿＿＿＿＿

**I．Information on hospital visit or medical aid for this incident stroke**

| I1 | Stroke onset time（i.e., the time that the patient looked normal last）： | | | |
| --- | --- | --- | --- | --- |
|  | Date：＿＿＿＿Year＿＿Month＿＿Day | □□□□ Year /□□Month /□□Day | | |
|  | Time：____Hour____Minute（24 hour time system） | | □□ Hour/□□Minute | |
| I2 | Was the stroke onset time exact (only one answer)?  1=Exact time（e.g.,08:42） 2=Estimated time（e.g., morning or 8～9 o'clock） 3=Unknown | | | □ |
| I3 | The time was found (by the patient him/herself or others)： | | | |
|  | Date：＿＿＿＿Year＿＿Month＿＿Day | □□□□ Year /□□Month /□□Day | | |
|  | Time：____Hour____Minute（24 hour time system） | | □□ Hour/□□Minute | |
| I4 | Was this incident stroke nighttime onset or daytime onset?  1=Nighttime onset 2=Daytime onset 9=Unknown | | | □ |
| I5 | Who found the symptoms?  1=Him/herself 2=Cohabitants 3=Witness (non cohabitants) 4=General Practioner in community 5=Other（indicate_____________________） 9=Unknown | | | □ |
| I6 | Was the patient awareness of that the initial symptom was a stroke?  1=Yes 2=No 3=Don't known what the disease is | | | □ |
| I7 | How did the patient seek a help when the initial symptom occur or after stroke onset?  1=Go to hospital by self 2=Phone to family 3=Seek neighbour's help 4= Seek stranger's help 5=Phone to GP for help 6=call 120/999 for help  7=Other（indicate_____________________） 9=Unknown | | | □ |
| I8 | How to seek medical service behavior after symptom onset？  1= Go to hospital by self 2= call 120/999 for ambulance  3= Wait for GPs at home 9=Unknown | | | □ |
| I9 | What was means of transport to the hospital after stroke onset?  1=Taxi 2=Private car 3=Bicycle, tricycle etc. 4=Ambulance(120)  5=Ambulance(999) 6=Ambulance (other) 7=Other______ 9=Unknown | | | □ |
| I10 | Was the stroke identified by ambulance doctor? (The question is applicable to the patient who was transferred by ambulance).  1=Yes 2=No 3=Not applicable 9=Unknown | | | □ |
| I11 | A.Hospital that the patient and family requested to ambulance's staff：_____________________________（Invsetigators must fill in.） | | | |
|  | Level of hospital（Collaborating centers is responsible for identification of hospital level and fill in, invsetigators mustn't fill in）  1=Tertiary hospital with qualification of thrombolysis 2=Tertiary hospital without qualification of thrombolysis 3=Secondary hospital 4=Local primary hospital/CHS centers 5=CHS stations 6= Individual clinic or private hospital | | | □ |
|  | B.Hospital where the patient was transferred by ambulance：_____________________________ (Invsetigators must fill in.） | | | |
|  | Level of hospital（Collaborating centers is responsible for identification of hospital level and fill in, invsetigators mustn't fill in）  1=Tertiary hospital with qualification of thrombolysis 2=Tertiary hospital without qualification of thrombolysis 3=Secondary hospital 4=Local primary hospital/CHS centers 5=CHS stations 6= Individual clinic or private hospital | | | □ |
| I12 | Date and time of hospital arrival：（24 hour time system） （99=Unknown） | | | |
|  | □□□□ Year /□□Month /□□Day□□ Hour/□□Minute | | | |
| I13 | If the time of stroke onset or hospital arrival was not exact, please estimate the time interval between stroke onset and hospital arrival.：  1= <2 hours 2= 2-6 hours 3= 6-12 hours 4= 12-24 hours  5= 1-3 days 6= 3-7 days 7= >7 days 9=Unknown | | | □ |
| I14 | Transfer time on the road of first-visit to a hospital after stroke onset：  1= <2 hours 2= 2-6 hours 3= 6-12 hours 4= 12-24 hours 9= Unknown | | | □ |
| I15 | A.First-visit hospital：_____________________________(Invsetigators must fill in.） | | | |
|  | B. Level of first-visit hospital（Collaborating centers is responsible for identification of hospital level and fill in, invsetigators mustn't fill in）  1=Tertiary hospital with qualification of thrombolysis 2=Tertiary hospital without qualification of thrombolysis 3=Secondary hospital 4=Local primary hospital/CHS centers 5=CHS stations 6= Individual clinic or private hospital | | | □ |
| I16 | Changes of patients’ symptoms from stroke onset to hospital arrival：  1=Worsened 2=Completely improved 3=Partially improved 4=Unchanged 9=Unknown | | | □ |
| I17 | Was thrombolytic therapy informed to the patient by a neourologist at first-visit hospital？  1=Yes 2=No 9=Unknown | | | □ |
| I18 | Did the patient receive the thrombolysis with t-PA?（The question is not applicable for patients with hemorrhagic stroke）  1=Yes 2=No 3=Not applicable 9=Unknown | | | □ |
| I19 | Reasons for not receiving thrombolytic therapy：（The question is not applicable for patients with hemorrhagic stroke）  1=Doctor told that time was overdue 2=Doctor told that patient was not suitable for thrombolysis 3=Patient or their families thought that the risk of thrombolysis was too large 4=Patient or family members thought that thrombolytic therapy was too expensive 5=Not applicable 9=Unknown | | | □ |

Reporter：＿＿＿＿＿＿ Reporting date：＿＿＿＿＿＿＿＿

Auditing physician：＿＿＿＿＿＿ Auditing date：＿＿＿＿＿＿＿＿
